# Upon the Evil Practices of Sundry Druggists in Neglecting the Regulations Enacted by the London College of Physicians in Their Pharmacopœia

**Published:** 1815-02

**Authors:** 


### *For the Medical and Physical Journal.*

### *Upon the evil Practices of sundry Druggists in neglecting the Regulations enacted by the London College of Physicians in their Pharmacopœia.*

FROM the age of Nicolaus Præpositus, of Tours, who wrote the first General Dispensatory, the compositions in which were principally taken from Mesue and from Nicolaus de Salerno, down to the last London Pharmacopœia, so many mistakes have arisen from the adoption of different formulas, and from the arbitrary presumption of chemists, that the executive, with a prudent attention to the public welfare, have sanctioned and enforced the regulations which the College of London has, at different times, judged proper to institute.

I am not discussing here the merits or demerits of the last London Pharmacopœia. I have perused, at different times, in your Numbers, some very appropriate strictures on some of its articles; but what will be your surprise to be informed, that any mischief can have ensued from those remarks, and that those very strictures have been confidently broached as adequate apologies for deviating from the orders of the College! Now, however criticism may have succeeded in detecting defects in some of its parts, no experienced man has ever denied, that, for the benefit of the community, it is essentially requisite to furnish the profession with some general standard of compounding medicine, by which, as the proclamation expresses it, all deceits, differences, and uncertainties, may effectually be prevented, and the art of medicine, at all times conjectural and replete with difficulties, may not be rendered more so, by the caprice and presumption of ignorant pretenders, whose quackery might strike at the stamina of the public health. It is for this purpose that his Majesty has thought fit, by the advice of his council, to give effect to the labours of the College, and "hath also charged and commanded all and singular apothecaries and others, whose business it is to compound medicine, &c. &c. within any part of his Majesty's Kingdom of Great Britain called England, &c. &c., that they, and every of them, after the publication of the Pharmacopœia of the London College, do not compound any medicine in any other manner than is directed by the College."

It is to be lamented, from the many alarming violations daily practised by chemists and druggists, that they are not *practically taught* that they are liable to be proceeded against "for such their contempt and offences, according to the utmost severity of the law." It really becomes a most serious evil, and calls aloud for redress, when the most consummate skill and experience are rendered nugatory and unavailing, by the unauthorised and spontaneous manner with which many druggists allow themselves to make up prescriptions; so that a medicine, thus compounded, shall have totally different properties to what its prescriber intended. This is not merely the case with minor druggists, but with houses of the first reputation, who supply our apothecaries with their medicines, and are permitted with impunity to *supersede the orders of the College.* The last London Pharmacopœia may have its exceptions, but these are at least innocent: let us not, by discarding it, draw down upon us the still greater evil of adopting the succedaneums of chemists—

> 'Tis better that we bear the ills we have,
> Than fly to others which we know not of.

If it were practicable to analyse every compound medicine before we ordered it, little injury might possibly accrue from the variety of private formulas assumed by different chemists, especially in *districts more remote from the vigilance of the College;* but, as this is not feasible, who, while he gropes in the dark, can prescribe without fear and trembling? If we take a cursory view of some of the articles of the last London Pharmacopœia, almost in the very first medicine, the citric acid, an article of great expenditure in private practice, most of the shops have a compound of citric and tartaric acid, some in equal parts. The last of these articles, you well know, is not half the price of the former. It is no excuse for the chemist to say, that the small quantity of neutral salt that the tartaric acid makes with the soda or potass in the saline draught, can do no harm. Admitting it were the case, is it to be endured that a chemist shall usurp the province of physician? is he to reason on the propriety of adopting succedaneums in medicine? who gave him a license to be a censor of the College? If this is connived at, a door is opened for abuse which is pregnant with the most fatal prospects, and threatens the lives of millions. If a chemist must exercise his ingenuity, let it be in licensed nostrums, not by surreptitiously introducing cheaper or more convenient succedaneums in prescriptions, by which the reputation of the prescriber and the health of the patient are made the victims. If, however, they are bent upon following up their antiquated prejudices, or more probably their own private interest and convenience, let them sue government for a charter to erect a College at York, which may be be made the standard for all the northern counties, in some of which there appears such a gross dereliction from the orders of the London College. I could mention many abuses, but what I shall here introduce will answer my purpose.

The Confectio Aromatica should contain nearly one quarter of Testæ præparatæ, and is therefore preferred by prescribers, on that account, in complaints where there is a laxity or redundant acidity of the intestines. But how are we to look for either antacid or absorbent properties from the simple Pulvis Aromaticus, or Capsicum with syrup? If no direct injury ensues, yet the value of the remedy and the accuracy of the prescription is impaired, because we take for *granted, at the time of prescribing, that it is made up according to the last orders of the College.*

A great part of the Sp. Ammoniæ is made from old forms in use a century back. Instead of volatile oil, which can only be dissolved in the genuine Sp. Ammoniæ, when the ammonia in the solvent is caustic, we now and then see an infusion of cloves, or other aromatics, mixed with Sp. Ammoniæ, made with Carbonate of Ammonia; a good reason why they cannot obey the orders of the College in using the Volatile Oil of Cloves, which would *float on the surface,* from its insolubility. It should contract besides an amber tinge by standing, from the oil of cloves. A certain knight of the pestle, who wanted braying in his own mortar, excused himself in deviating from the prescribed method of the College, which requires the use of Caustic Ammonia and Volatile Oil in the Sp. Ammoniæ, by gravely assuring his hearers, that neither the Sp. Ammoniæ or the Sp. Ammoniæ Aromat. were ever used internally, never given in the stomach!!! You will no doubt be eager to record so egregious a discovery, because the profession has been blundering all along, according to this gentleman, in administering them even to infants, in women in hysterical affections, and in both sexes, as a stimulant and antispasmodic, with incredible advantage. Of course, whatever objection is made to the composition of the Sp. Ammoniæ, obtains equally when applied to all those medicines that contain it, as the Sp. Ammoniæ Fœtid., Tinct. Valerian Ammoniat., and Tinct. Guaiac. Ammoniat.

But is this strange, when I have been credibly informed that the Liquor Ammoniæ and the Liquor Ammoniæ Carbonatis are in some shops used indiscriminately? I have examined with nicety similar drugs in the metropolis, without corresponding inaccuracies. I have never, for instance, on analysing the neutral salts, found them so sophisticated or impure, such as the Nitras and Carbonas Potassæ, the Carbonas bonas and Sulphas Magnesiæ, the last of which seldom yields in the experiments and specimens which I have *recently* adopted, the proper proportion of magnesia on the addition of Carbonas Potassæ. Indeed an accurate inspection of the chrystallization easily points out the presence of a cheaper salt, the Sulphat of Soda. I have heard of much confusion, and sometimes serious mischief, which has arisen from confounding the Liquor Antimon. Tartariz. with the Vinum Antimonii Tartariz. Nothing but sheer carelessness can apologise for this neglect, and, where such instances can be proved, they should be made objects of the penalty. I say nothing of the custom of compounding the Sp. Ammon. fœtid. with a simple admixture of Spir. Ammoniæ and Tinctur. Assafœtid. or of the æthers, and especially the Æther Sulphuricus, which is notoriously in many cases of inferior purity to that ordered by the College, as is indicated both by its specific gravity, and the precipitate on adding a solution of muriate of barytes.

These abuses, I believe, are not peculiar to any quarter. If a druggist should choose to make his Pulv. Scammon. Composit. or his Tinct. Aloes Composit. the first without the hard extract of jalap, and the last with half the proportion of aloes, according to the Edinburgh College, are such aberrations to be accounted venial? I am not contending which is the best Pharmacopœia, that of London, Edinburgh, or Dublin, or that any one of them is free from objection; but that for accuracy's sake there must be *only one acted upon in the same place, and that one in England we know by the law of the land to be the London Pharmacopœia*. If some of your correspondents will favour the public with their researches in other districts, it may possibly *call the attention of the College* to the many very flagrant abuses, which draw down unnecessary opprobrium on our art, and inflict a serious blow on the health of the community.

*Yorkshire.* *A regular Physician.*

